# Clinical and morphological effects of hyperbaric oxygen therapy in patients with interstitial cystitis associated with fibromyalgia

**DOI:** 10.1186/s12894-019-0545-6

**Published:** 2019-11-05

**Authors:** Gerardo Bosco, Edoardo Ostardo, Alex Rizzato, Giacomo Garetto, Matteo Paganini, Giorgio Melloni, Giampiero Giron, Lodovico Pietrosanti, Ivo Martinelli, Enrico Camporesi

**Affiliations:** 1 Environmental and Respiratory Physiology Laboratory, Department of Biomedical Sciences, University of Padova, Padova, Italy; 2 Unità Operativa di Urologia, Azienda Ospedaliera Santa Maria degli Angeli, Pordenone, Italy; 3 ATIP Centro di Medicina Iperbarica, Padova, Italy; 4 Department of Statistics, Harvard School of Medicine, Boston, MA USA; 5 OTI Services, Centro di Medicina Iperbarica, Venezia, Italy; 6 TEAMHealth Research Institute, TGH, Tampa, Florida USA

**Keywords:** Hyperbaric medicine, Interstitial cystitis, Fibromyalgia

## Abstract

**Background:**

Interstitial Cystitis (IC) is a debilitating disorder of the bladder, with a multifactorial and poorly understood origin dealing with microcirculation repeated damages. Also Fibromyalgia (FM) is a persistent disorder whose etiology is not completely explained, and its theorized alteration of pain processing can compromise the quality of life. Both these conditions have a high incidence of conventional therapeutic failure, but recent literature suggests a significant beneficial response to Hyperbaric Oxygen Therapy (HBOT). With this study, this study we evaluated the effects of HBOT on quality of life, symptoms, urodynamic parameters, and cystoscopic examination of patients suffering from both IC and FM.

**Methods:**

We structured an observational clinical trial design with repeated measures (questionnaires, urodynamic test, and cystoscopy) conducted before and 6 months after a therapeutic protocol with hyperbaric oxygen for the treatment of patients suffering from both IC and FM. Patients were exposed to breathing 100% oxygen at 2 atm absolute (ATA) in a multiplace pressure chamber for 90 min using an oro-nasal mask. Patients undertook a cycle of 20 sessions for 5 days per week, and a second cycle of 20 sessions after 1 week of suspension.

**Results:**

Twelve patients completed the protocol. Changes after HBOT were not significant, except for hydrodistension tolerance (mean pre-treatment: 409.2 ml; mean post-treatment: 489.2 ml; *p* < 0.05). A regression of petechiae and Hunner’s ulcers was also noted 6 months after the completion of HBOT.

**Conclusions:**

Our study showed no improvement of symptoms, quality of life, and urodynamic parameters, except for hydrodistension, and a slight improvement in cystoscopic pattern. However, to date, we could not demonstrate the significance of overall results to justify the use of HBOT alone in patients with IC and FM. This observation suggests that additional studies are needed to better understand if HBOT could treat this subset of patients.

**Trial registration:**

NCT03693001; October 2, 2018. Retrospectively registered.

## Background

Interstitial Cystitis (IC) is a rare, chronic, and disabling condition of the bladder that mainly affects females [1–3]. The specific etiology of IC is currently unknown but seems multifactorial, with interactions among autoimmune, neuroendocrine, allergic, and infectious pathways [4, 5]. Theories suggested that IC could derive from an abnormally increased number of mast cells, or could be related to an alteration of the glycosaminoglycan layer protecting urothelium from urine [6]. The activation of the inflammatory response induces alterations in the deep layers of bladder, such as fibrous substitution of the muscular tunic, thinning and discontinuity of the mucosa layer, capillary proliferation, and blood vessel degeneration [7].

Initial presentation of IC is subtle. The possible presence of infection (due to urothelium damage), an increase in void frequency, and a pain resistant to analgesia are early symptoms that make the diagnosis more challenging because of overlapping with those present in bacterial cystitis and several other diseases [8]. Unfortunately, patients spend about 5 to 10 years and a mean of 8 consults from different specialists before a correct diagnosis [9], while recurrent inflammation results in scar tissue development. The subsequent reduction of both bladder compliance and capacity, in conjunction with the gradual loss of functionality, determine chronic urinary tract symptoms [10], thus prompting cystoscopy and urometry that finally make the late diagnosis. Since the primary cause of IC is still hypothesized, conventional treatments – such as physical therapy, antidepressants, pentosan sulfate, immunosuppressants, intravescical therapy with lidocaine heparin and bicarbonate, and surgery [11] – mainly aim to alleviate symptoms. The effectiveness of most treatments does not exceed 60%, and symptoms return even after a period of improvement or recovery [9].

A pilot study showed that 76% of patients with histologically confirmed IC have another medical condition, such as Fibromyalgia Syndrome (FM), Chronic Fatigue Syndrome, and Irritable Bowel Syndrome [12]. FM is a persistent and debilitating disorder that compromises the quality of life, affecting 2–4% of the population with a 9:1 female to male ratio [13]. There is no agreement on the specific etiology of FM, even if some authors suggest that an abnormal brain activity regarding pain processing could be the leading cause [13]. Patients suffering from FM typically present with a triad of widespread chronic pain of long duration (> 3 months), sleep disturbance, and fatigue. Nonetheless, the possible association with other key symptoms such as allodynia, hyperalgesia, general muscular tension, nerve pain, cognitive impairment, and mood disturbance makes the diagnosis quite challenging because of several overlaps with other rheumatologic conditions [13, 14]. All these symptoms are included in the 2010 Fibromyalgia Diagnostic Criteria published by the American College of Rheumatology [15, 16].

Several integrated programs were proposed for FM, mainly targeting symptoms management using both pharmacological and physical exercise or behavioral therapy, but there is no consensus about these treatments, that have still limited effectiveness [13].

Recently, an increasing amount of literature suggested the efficacy of Hyperbaric Oxygen Therapy (HBOT) in patients with IC or FM. For instance, van Ophoven and colleagues reported an improvement of symptoms and bladder capacity in patients affected by IC and treated with HBOT [5], results confirmed also by Tanaka in patients with a form of IC resistant to conventional therapy [4]. On the other side, Yildiz and colleagues found a significant reduction of Visual Analogue Scale scores in patients affected by FM and a significant increase in pain threshold [17]. Moreover, the work of Efrati and colleagues demonstrated a decrease in symptoms of FM and positive changes in brain activity [13], concluding that HBOT plays an important role in FM management. However, no study has investigated the possible role of HBOT in patients affected from IC associated with FM (IC/FM) so far.

The aim of our study was to investigate the response of IC/FM patients subjected to HBOT. In detail, we evaluated the effectiveness of HBOT in IC/FM refractory to conventional therapy, focusing on changes in quality of life, pain modulation, modifications in bladder endoscopic and urometric patterns.

## Methods

### Subjects

**Fig. 1:**
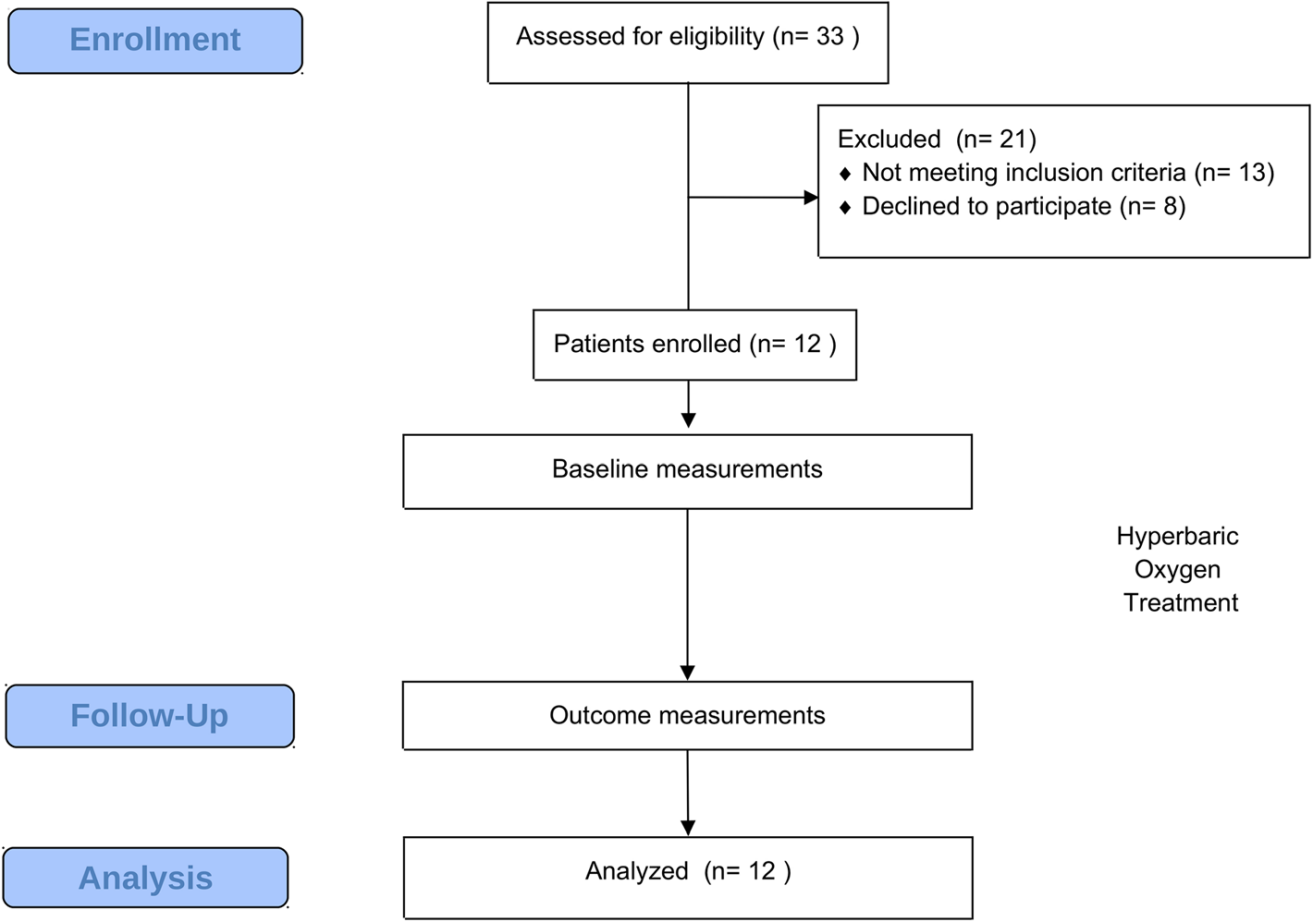
Flow-chart of experimental design. Eligibility and recruitment of patients. Details are reported in the text

Patients were enrolled and considered eligible for the study after a medical screening carried out at the ATiP Center of Hyperbaric Medicine (Padova, Italy), in order to exclude possible contraindications to HBOT (Fig. 1). No incentives were offered to increase the enrollment and compliance to the study. The inclusion criteria were: (a) pain in bladder filling that improves with urination; (b) pain (suprapubic, pelvic, urethral, vaginal, or perineal); (c) presence of glomerulation (grade II/III) (or bleeding +/− at the cystodistension) and positive histologic findings at biopsy [18]; (d) reduced capacity; (e) increased visceral sensitivity; (f) normal or reduced compliance; (g) symptoms refractory to conventional therapy; and (h) diagnosis of FM according to the 2010 American College of Rheumatology guidelines [15, 16]. These criteria were based on those of the European Society for the Study of Interstitial Cystitis (ESSIC), including patients with an ESSIC disease staging ≥2C [18].

The exclusion criteria were: (a) pregnancy (diagnosed or within the previous year); (b) age less than 18 years; (c) benign or malignant bladder tumors; (d) radiation cystitis; (e) symptomatic bladder diversions; (f) herpes in active phase; (g) bladder and urethral stones; (h) urinary frequency less than 10 times a day; (i) presence of symptoms less than 12 months; and (l) bladder capacity > 400 ml with no sensitive urgency.

### Experimental protocol

The experimental protocol received the approval by the local Human Ethical Committee (n° HEC-DSB 07/16) of the Department of Biomedical Science at University of Padova and adhered to the principles of the Declaration of Helsinki. Patients involved in the study read and signed an informed consent, were free to renounce the study at any time, and every precaution was taken to protect their privacy. All the patients were informed about the methods and aims of the study.

We structured an observational clinical trial design with repeated measures conducted before and 6 months after a therapeutic protocol with hyperbaric oxygen for the treatment of patients suffering from IC/FM. The authors confirmed that all ongoing and related trials for this intervention were retrospectively registered in the clinicaltrials.gov registry (NCT03693001). This study was not registered before enrollment of participants started because IC is an approved indication for HBOT in Italy and HBOT sessions were already planned in the therapeutic schedule of the enrolled patients. The whole study, comprehensive of recruitment and follow up after the experiment, took place between January and September 2018. This period seemed adequate for an evaluation over time of the treatment results on the most relevant symptoms of the pathology, i.e. pain, frequency of urination, urgency and evaluation of bladder capacity [1, 5]. Patients were exposed to breathing 100% oxygen at 2 atm absolute (ATA) in a multiplace pressure chamber (Galeazzi, Zingonia, Italy) for 90 min using an overboard demand regulator while breathing through an oral-nasal mask. Each patient undertook a daily cycle of 20 sessions, 5 days a week. After 1 week of suspension, a second identical cycle of 20 sessions was performed.

The primary outcome was the modification in symptoms, assessed through several questionnaires that were administered to patients before (PRE) and 6 months after (POST) HBOT: (a) three-day voiding diary, (b) widespread pain index (WPI), (c) symptom severity scale (SSS), (d) Pelvic pain, Urgency and Frequency symptom scale (PUF), and (e) O’Leary-Sant questionnaire.

### Questionnaires

#### Three-day voiding diary

It is a sheet for each 24-h period. Patients specified their bedtime and wake-up time directly in the upper part of the sheet. Later, they started recording all fluid intake (i.e., the total amount of fluids drank during a given time period) and urinary events (i.e., the amount of urine voided each time over a 24-h period). Moreover, the voiding diary presents specific fields to indicate either the amount of urine drained via catheter and/or each time the pad was changed. Patients write each time they had involuntary urine loss (even a small amount). As final outcomes, mean and maximum urine volume were considered.

#### Widespread pain index

WPI is a clinical diagnostic criterion proposed for patients with FM that do not rely on counting tender points [15]. It considers not only pain but also other FM-related symptoms assessing their severity [15]. Physician asked the patient to indicate the location of any pain experienced during the week before the exam. As a result, WPI pointed out a total amount ranging from 0 to 19 points corresponding to the possibly-painful 19 body areas (i.e., areas of the shoulders, arms, hips, legs, jaws, chest, abdomen, back, and neck) [14].

#### Symptom severity scale

SSS focuses on 3 physical symptoms, as well as somatic symptoms in general [16]. In detail, symptoms are measured on the basis of a 0–3 severity scale considering fatigue, waking unrefreshed, and cognitive symptoms. The investigated period is the week before the questionnaire administration [14]. Later, the items are combined into a 0–12 scale assessing the somatic symptoms in general. The greater the amount, the more severe the symptomatology.

#### Pelvic pain, urgency and frequency symptom scale

Pelvic pain, Urgency and Frequency scale (PUF) is an eight-item questionnaire, largely used to evaluate symptoms of IC. It is organized in two subscales: symptom severity and level of bother. Total scores range from 0 to 35 (symptom subscale 0–23 and bother subscale 0–12). The higher the scores, the more severe the level of symptoms [19]. Scores > 12 indicate significant symptoms; scores of ≥15 have an 84% sensitivity in diagnosing IC based on positive potassium testing, which indicates abnormal permeability of bladder epithelium [1]. Also, the PUF was previously reported in persons with FM [20].

#### O’Leary-Sant index

The O’Leary-Sant index – also named Interstitial Cystitis Symptom Index (ICSI) – was proposed as a uniform outcome measure in IC [21]. The Interstitial Cystitis Problem Index (ICPI) was later developed from the ICSI as a corresponding problem index [22]. It measures the symptoms of the lower urinary tract and their influence on quality-of-life in subjects with IC. Test-retest reliability analysis and validation via administration to IC patients and asymptomatic controls resulted in a questionnaire with 8 items outlining two indexes: the symptom and the problem index [21, 23].

### Cystoscopy, hydrodistension, and urodynamic evaluation

As secondary outcome, modifications in bladder endoscopic and urometric patterns were searched. Patients were assessed before (PRE) and 6 months after (POST) HBOT through cystoscopy, hydrodistension, and urodynamic examination. Hydrodistension was performed without sedation, applying a maximal filling pressure of 20–25 mmHg for 10 min. Petechiae were graded at PRE according to Nordling [24], and the improvement in lesions was defined as a downgrade found at POST. Urodynamic testing allows recording of bladder sensitivity, capacity, and compliance as well as urethral and detrusor activity during filling. PRE and POST cystoscopy, hydrodistension, and urodynamic testing were performed by the same urologist, and urodynamic evaluation followed current International Continence Society standards.

### Statistical analysis

Data were coded on a master sheet using a LibreOffice Calc spreadsheet (ver. 6.0.1.1, The Document Foundation, Berlin, Germany). A Two-tailed t-test for dependent means was used to analyze differences between pre and post-treatment means of questionnaires and quantitative data from Urodynamic testing, with a significance level of .05. Qualitative data from cystoscopy were analyzed descriptively.

## Results

Thirty-three patients were initially enrolled for the study, and no contraindications to HBOT were found after medical screening. After application of inclusion and exclusion criteria, twenty patients were considered eligible for the study. However, 8 patients withdrew during the study (1 had problems with compensation maneuvers in the chamber; 3 because of the long travel needed to reach the hyperbaric facility; 4 did not provide a reason), and twelve patients concluded the study (M = 1; F = 11; mean age ± SD: 57 ± 10.57 years; mean duration of illness ± SD: 10,6 ± 9,33 years).

HBOT did not result in a statistically significant improvement both in questionnaires and in urodynamic testing, except for hydrodistension (mean pre-treatment: 409.167 ml; mean post-treatment: 489.167 ml; *p* < .05) (Table 1).

**Table 1:** Changes after hyperbaric oxygen treatment

	PRE	POST	*P* value
WPI	7.08 ± 5.14	5.75 ± 4.00	.46
SS	8.16 ± 2.88	7.50 ± 4.25	.60
PUF	22.83 ± 6.20	20.41 ± 6.69	.24
OS	13 ± 5.86	11.08 ± 5.17	.12
Mean urine volume (ml)	193.83 ± 89.61	201.27 ± 91.16	.75
Max urine volume (ml)	425.00 ± 257.30	383.33 ± 187.9	.47
Hydrodistension (ml)	409.16 ± 196.30	489.16 ± 149	.04
1st desire	103.50 ± 48.61	114.75 ± 37.33	.31
Strong desire	247.33 ± 99.79	245.41 ± 113.7	.91
Compliance	41.85 ± 27.06	44.18 ± 24.99	.74
Cystomanometric Capacity (ml)	318.10 ± 126.50	310.33 ± 133.5	.73
Urethral functioning (tension)	68.41 ± 25.69	75.33 ± 23.17	.47

After 20 HBO treatments, no statistically significant changes in assessed items were detected, except for hydrodistension (*p* < .05). *WPI* Widespread Pain Index, *SS* Symptom Severity score, *PUF* Pelvic Pain and Urinary Urgency Frequency, *OS* O’Leary Sant symptoms and problems index. Hydrodistension: derived from cystoscopy; 1st desire, voiding pain, compliance, cystomanometric capacity, and urethral functioning: derived from urodynamic testing

Cystoscopy performed after the treatment detected a reduction of petechiae and a regression of Hunner’s ulcers (initially detected in only one patient), while glomerulations remained the same (Table 2).

**Table 2:** Alterations observed in patients during cystoscopy before and 6 months after HBOT

	PRE	POST
Petechiae	11 patients	7 patients
Glomerulations	8 patients	8 patients
Hunner’s ulcers	1 patient	no patients

After HBOT, there was a reduction of petechiae among treated patients, while no change was recorded regarding glomerulations. Of note, the only one patient presenting with Hunner’s ulcers experienced a regression of the lesion.

## Discussion

HBOT is emerging as a novel treatment in patients with IC and FM, as suggested by recent experiences in both the urologic and rheumatologic fields. In fact, hyperbaric oxygen is known to increase anti-inflammatory defenses over time and to promote neovascularization [25]. These mechanisms have been recently confirmed by Minami, who reported a reduction in H2O2-induced inflammation, edema, and fibrosis on the bladder of mice treated with HBOT [26]. On a molecular level, Thom demonstrated mobilization of bone marrow stem cells in response to hyperoxia, to hyperoxia and concluded that species that oxidative stress is the fundamental key mechanism of action of HBOT, exerting its effects on transcription through reactive oxygen and nitrogen species production over several treatments [27]. Similarly, Minami and coll. demonstrated a decrease in mRNA expression of inflammatory biomarkers and an increase in endothelial nitric oxide synthase (eNOS) after HBOT [26]. Overall, HBOT results in enhanced perfusion and wound healing in ischemic tissues [25, 27].

HBOT is now widely used in urology for the treatment of radiation cystitis, cyclophosphamide-induced cystitis, emphysematous cystitis, or pelvic radiation disease, and the interest in treatment IC is currently growing [28]. The molecular mechanisms of IC development are still not well known, but current literature identifies a plausible cause in microcirculation. For instance, Tamaki and coll. suggested that an important pathophysiology way could result from a reduced blood flow in bladder’s walls during its filling phase [29]. Concordantly, Lee J.D. and Lee M. verified that, in the bladder of IC patients, the expression of hypoxia-inducible factor-1α is increased both in muscle and urothelium of bladder, and Vascular Endothelial Growth Factor (VEGF) expression is higher in umbrella cells (apical cells) [6]. Both these studies could explain a hypoxic/ischemic damage, and therefore a possible useful role of HBOT in IC, as previously demonstrated on an ischemia/reperfusion model on muscle mice by Bosco and coll. [30].

As hypothesized by Efrati and coll., HBOT induced an improvement of symptoms in patients with FM by increasing brain oxygenation, promoting neoangiogenesis and restoring normal patterns of pain processing [13].

Besides these recent evidences that favor HBOT in patients with IC and FM, no literature is available about such a treatment in patients with both the diseases, probably because of the rarity of this overlap in the general population.

Surprisingly, our results showed an isolated statistically significant improvement in hydrodistension, but no significant improvement in scores evaluating symptoms and life quality or other parameters at urodynamic testing (Table 1). These findings prompt several considerations, since no previous study has investigated the usefulness of HBOT in IC/FM patients.

With regard to FM, these findings do not support previous works that used HBOT to treat patients with FM alone [13, 17]. On the other hand, from the perspective of IC, our sample mostly included patients with non-ulcerative IC (11 out of 12) and our results are consistent with those of van Ophoven [5], Tanaka [4], and Wenzler [31] that demonstrated few effects on this subset of patients. Overall, this inconsistency may be due to the considerable delay in diagnosis of an IC/FM overlap and the conventional therapy administration, thus leading to resistance or failure to treatments. Another possible explanation for these results resides within the complicated medical management of these patients. In fact, since patients with FM have altered pathways of pain processing, an overlap with IC could probably worsen symptoms, and HBOT alone may be not sufficient to treat an overlap of both these diseases.

In IC, urothelial alteration and deficiency of glycosaminoglycans may lead to a thinning of the mucosa up to the presence of Hunner’s ulcers and/or fascicular fibrosis [29]. Cystoscopy usually documents these alterations by highlighting a bladder wall with inflamed areas, hemorrhagic areas, and fibrosis of the inner mucosal lining. If compared with non-ulcerative IC, Hunner’s ulcers are characterized by histological findings of pancystitis, increase in plasma cells, expansion of clonal B-cells, and epithelial denudation [32]. According to our findings, glomerulations remained the same but there was a reduction of petechiae in most of the patients, thus suggesting possible improvement in oxygenation of apical tissues (Table 2). Expression of VEGF was not among the goals of our study but, according to literature, a reduction in petechiae could be linked with a reduction in VEGF expression in apical cells [6]. We also assisted to a regression of Hunner’s ulcers after HBOT, a finding that matches those observed in recent studies by Tanaka [4] and by Wenzler [31] (Table 2).

The results of this study are promising but are subjected to a number of limitations. First, given the rarity of IC/FM overlap, we were able to enroll only 20 patients and 12 completed the study. Given the limited number of participants and the absence of a control group, results should be interpreted carefully. However, our sample size is greater than or similar to those of others previously cited studies, and a wider study – possibly multicentric – could help the scientific community to overcome this impasse. Second, since FM can overlap with several other conditions, the patients could have suffered from other diseases than IC and FM. In the future, a more precise stratification could be useful to draw conclusion on a larger sample of patients. Third, this study evaluated HBOT alone on patients, without tracking any other interventions such as education therapy, diet, or psychotherapy. Since the key of complex syndromes management resides in a multidisciplinary approach, we think that future trials should evaluate HBOT not alone but together with conventional treatments, in order to quantify its usefulness as an adjuvant therapy.

## Conclusions

Patients with both IC and FM suffer from a complex and rare syndrome with a high level of therapeutic failure. According to literature, HBOT seems to be clinically effective in treating these two diseases when separated. Our study showed a statistically significant improvement of hydrodistension alone, but no significant improvement in symptoms, quality of life, and other urodynamic parameters in this subset of patients. Moreover, HBOT resulted in an improved cystoscopic pattern, with a healing of petechiae and Hunner’s ulcers. Anyway, our results cannot justify HBOT alone in IC/FM patients. This is a spur to expand the sample and promote trials in the same field to better understand how HBOT could treat this rare subset of patients, especially as an adjuvant to conventional treatments.

## Data Availability

Not Applicable.

## Abbreviations

ATA: Atmospheres Absolute
FM: Fibromyalgia
HBOT: Hyperbaric Oxygen Therapy
IC: Interstitial cystitis
ICPI: Interstitial Cystitis Problem Index
ICSI: Interstitial Cystitis Symptoms Index
PUF: Pelvic pain, Urgency and Frequency scale
SSS: Symptom Severity Scale
VEGF: Vascular Endothelial Growth Factor
WPI: Widespread Pain Index
